# Fault lines in forensic medical toxicology in Ireland exposed through replies of pathologists and coroners to anonymous questionnaires

**DOI:** 10.1186/2193-1801-3-531

**Published:** 2014-09-16

**Authors:** William P Tormey, Ingrid Borovickova, Tara M Moore

**Affiliations:** Biomedical Sciences, University of Ulster, Coleraine, BT52 1SA Northern Ireland; Department of Chemical Pathology, Beaumont Hospital, Dublin 9, Ireland

**Keywords:** Coroners, Pathologists, Questionnaires, Toxicology, Ireland

## Abstract

The attitudes and experiences of pathologists and coroners to the provision of biochemical forensic toxicology in the Republic of Ireland were determined using separate questionnaires to each group anonymously. Replies were received from 36/88 (41%) of pathologists and 19/71 (27%) of coroners. 37% of coroners considered that histopathologists give an adequate opinion in forensic toxicology yet 58% of pathologists reported that they did not have adequate access to expert medical interpretative toxicological opinion. For drug-drug interactions and metabolic diseases, 69% of pathologists were unhappy with the processes and 68% of coroner replies did not know if vitreous samples were used appropriately. There is a clear requirement for retraining of coroners and for the appointment of medical toxicology expertise to improve the quality of service for coroners.

## Introduction

The rationale for developing a National Institute for Forensic Toxicology in Ireland was published (Tormey 2013). It served as a critique of the toxicology service for coroners in Ireland and outlined detailed recommendations for corrective actions. Because the paper was published by Open Access, it was widely read by government officials and pathologists involved in the coronial service. By June 22 2014, there were 878 total accesses listed in the article metrics by Springer. Despite this and an acceptance of the validity of the professional governance issues raised, there has been inertia with regard to change management. The State Laboratory in Kildare is used for coronial toxicology and has widened its analytical service but has no formal arrangement with a hospital laboratory for routine biological analytes including, glucose, urea, creatinine, lactates, ketones, insulin and proinsulin. Laboratory reports for pathologists have no medically qualified toxicology input and interpretation is offered by an analytical scientist usually by quoting Baselt’s Textbook (Baselt 2011). This is an unsatisfactory governance structure which must be reformed. The State Laboratory accreditation is ISO/IEC 17025. 2005 2nd Edition and these are the general requirements for testing and calibration laboratories. By contrast, medical laboratories are assessed against ISO 15189 standard in developing their quality standard systems. Beaumont Hospital was formerly associated with the coroners’ service and was accredited by Clinical Pathology Accreditation (UK). Therefore there is a reporting hiatus for medical cases at the State Laboratory which is ignored.

Service user satisfaction is a conventional measure. There is no published information in Ireland on the requirements and attitudes of pathologists performing autopsies and the coroners receiving reports. Thus appropriate questionnaires were designed to consult each group and fill the information gap to facilitate change management (Boynton 2004).

## Methods and results

A separate six item questionnaire was sent to all practicing 88 histopathologists and 4 forensic pathologists listed in the Irish Medical Directory for 2013 (Irish Medical Directory 2013). A different six item survey was sent to all 72 coroners and deputy coroners listed on the Irish coroners service website (http://www.coroners.ie/en/CS/Pages/Coroner%20Contact%20Details). An explanatory letter was included with a stamped addressed envelope and there was no identifying mark on the paper questionnaire to preserve anonymity.

### Pathologists

The text of the explanatory letter to pathologists read:-

*“In coroner’s cases, the final interpretative report for the court is collated by the dissecting pathologist. The purpose of the survey is to garner the opinions of pathologists who collate the final autopsy report for the coroner’s courts on forensic biochemistry services supplied at present. Please circle the relevant Yes/No/Don’t Know at the end of each query.”*

The six questions with the replies to each question are listed. Despite none being requested, there were a small number of narrative comments linked to specific questions written on the returned questionnaires.

1. Do you have access to expert medical opinions on forensic toxicology values on drugs and metabolism when you collate your report for the coroner?

Yes 11 (30.6%). No 21 (58.3%). Don’t Know 4 (11.1%).

Narrative comments:

- “Interpretation as per State Laboratory report”
- “Other than what is on the State Laboratory report”
- “State Lab detailed report”
- “Not readily available at present.”
- “Senior chemists in State Laboratory used.”
- Would you prefer a multidisciplinary group meeting (MDM) of histopathologists, chemical pathologists and toxicologists to be available to issue, formal advice to the dissecting pathologist and therefore the coroner? Forensic toxicology cases would then be treated in the same way as complex hospital cases.

Yes 20 (55.5%). No 10 (28%). Don’t Know 6 (17%).

Narrative comment:

- “Just chemical pathologist and/or toxicologist”
- “So far not required. Have attended many inquests and never a need to call a chemical pathologist or toxicologist so far”
- Do you think that drug/drug interactions and metabolic diseases such as diabetes/ketosis are adequately processed at present?

Yes 2 (5.5%). No 25 (69%). Don’t Know 9 (25%).

Narrative comment:

- “Would value help interpreting”
- Do you think a National Institute for Forensic Toxicology should be set up to facilitate improvement of standards in the coronial system in medical toxicology cases?

Yes 23 (64%). No 3 (8%). Don’t Know 10 (28%).

Narrative comment:

- “If you can find funding under current resource restraint”
- “Sufficient number of cases to warrant resource?”
- “Not relevant – the vast majority of my cases are exclusively perinatal.”
- Would you like to have easy access to an MDM group for some of your coroner’s cases?

Yes 29 (80%). No 7 (19%). Don’t Know 0.

Narrative comment:

- “Just chemical pathologist and/or toxicologist”
- Ideally, should a National Institute for Forensic Toxicology cover the whole island of Ireland and both jurisdictions?

Yes 16 (44%). No 5 (14%). Don’t Know 15 (42%).

Narrative comment:

- “Perhaps might allow economic case”

### Contrasting long narratives from two pathologists

There were two long narrative replies additional to those above. These show contrary views, insights and expectations of the roles of laboratory scientists in contrast to medical consultant chemical toxicology expertise.

*“The State Laboratory service provides us with an excellent service including detailed reports and references to support us in our interpretation of values drug levels etc. In seven years experience as a consultant performing 80 to 90 autopsies annually I have not required advice other than that provided by the State Laboratory. They are available to discuss results by telephone, add extra tests if additional levels are requiredand are generally extremely helpful. I have had several cases of diabetic ketoacidosis and they have managed to outsource testing for me for beta hydroxybutyrate etc. I do not have a requirement for additional support as long as the current service remains in place. However I realise that the day may come where an unusual query will arise but hopefully the State Laboratory will be able to advise. I am concerned re the new “legal highs” and whether the State Laboratory is testing for them currently. There are so many compounds in the UK market that it is only a matter of time before we see deaths here as a consequence but I hope the State Laboratory is up to speed on this. I intend to ask them the next time I am talking to them.”*

By contrast another pathologist wrote: *“While I would like access to toxicology expertise, this should be through the coroners office and not an MDT setting- ie equivalent to a toxicology consult. I think that this would be rarely required and only deal with the Republic of Ireland jurisdiction”.*

### Coroners

The explanatory letter to coroners and deputy coroners read:-

*“Services for coroners should be reviewed and updated regularly. The Department of Justice and Equality is likely to present a Coroner’s Bill to the Oireachtas soon. I have a specific interest in forensic toxicology. This summer, a protocol for the introduction of a National Institute for Forensic Toxicology was published (copy enclosed). It is important to establish overall opinions of coroners regarding what they would prefer in the area of biochemical toxicology. Please circle the relevant answer Yes/No/Don’t Know at the end of each query.”*

The six questions were:-

1. Do you want a specialist medically qualified group to be available to provide an expert interpretative services for the coroner’s service?

Yes 14 (74%). No 3 (16%). Don’t Know 2 (10%)

2. Is the present service satisfactory?

Yes 7 (37%). No 7 (37%). Don’t Know 5 (26%)

Narrative comment:

- “As far as my requirements go”
- Do you consider the advice on potential drug-drug interactions to be satisfactory at present?

Yes 7 (37%). No 7 (37%). Don’t Know 5 (26%).

4. Do you consider the current use of vitreous samples for diagnosis of metabolic disorders such as diabetes, ketosis, electrolytes etc to be appropriate?

Yes 5 (26%). No 1 (5%). Don’t Know 13 (68%).

5. Do you have expert advice available to you on medical toxicology at present?

Yes 6 (31%). No 10 (53%). Don’t Know 3 (16%).

Narrative comment:

- “As incorporated in the toxicology report”
- “Whenever this is necessary pathologists will procure same.”
- Do you consider the opinions of specialist histopathologists adequate in forensic biochemistry and toxicology cases?

Yes 7 (37%). No 5 (26%). Don’t Know 7 (37%).

Narrative comment:

- “Unless I am told they are not fit for purpose”

Overall, completed survey replies were received from 36 pathologists with 4 more declaring that they no longer performed autopsies giving an adjusted reply rate of 36/88 (41%). There were 19 replies from coroners with another stating that he was a solicitor and not a doctor so could not reply. This leaves an effective rate of 19/71 (27%). Because of the context of the necessity for uniformity of governance in forensic medicine, these low response rates do not invalidate the data.

## Discussion

The volumes of known deaths from medical misadventure are high. In 1999, there were 108,000 deaths from adverse drug reactions after administration of FDA-approved drugs in USA (http://www.rightdiagnosis.com/m/medical_misadventure/deaths.htm). Pharmacogenomics and drug interactions are increasing recognised and will play a part in the future in death investigations (Lam et al. 2014). Drug toxicities are common in western societies and the coronial system must act as a societal watchdog (Pilgrim et al. 2011). Therefore best practice is important.

The coroner service should be reasonably uniform in its governance and performance. Pathologists would be expected to have a relatively uniform experience and opinion of toxicology services if the provision was fit for purpose. Increasing specialisation in training programmes in medicine makes it unlikely that histopathologists as a group would retain expertise in toxicology and metabolic disorders to safely deal with non-standard cases. In parallel, analytical scientists are unlikely to be sufficiently skilled in medicine to provide a consultative service especially in the absence of regular exposure to an institutional multidisciplinary case meeting in the presence of appropriate medical expertise.

The replies from pathologists to all questions involved in interpretation, medical and toxicological expertise show a clear majority dissatisfied with the status quo and in favour of access to an expert group under the guise of a multidisciplinary group meeting or a National Institute for Forensic Toxicology. Some narratives show a lack of insight into other medical specialities. The method anonymity enhances the validity of the study but limits the percentage returns because reminders could not be targeted at those who failed to reply.

Support for and against a link with Northern Ireland was evenly balanced even though it is likely to be the most efficient way of dealing with a small number of cases likely to need expert advice.

The answers to the drug interactions and metabolic diseases questions in both surveys show the inadequacy of current practices in these areas. Sudden death is a feature in both diabetes and epilepsy and hypoglycaemia or electrolytes disorders as precipitating causes are likely to be missed. Vitreous sampling is not common and it is likely that many metabolic disorders are missed as a consequence.

Some of the narrative comments show that knowledge of the role and expertise of medical toxicologists and chemical pathologists is lacking. That 37% of coroners consider that histopathologists give an adequate opinion in forensic toxicology may have unfortunate consequences. 58% of pathologists reported that they did not have adequate access to expert medical interpretative toxicological opinion. Thus it is not surprising that there are reports of cases in Ireland where incorrect outcomes of inquests has resulted from inadequate pathology reports due to lack of chemical toxicology clinical expertise (Tormey 2012a, [b]).

These results confirm the necessity for appropriate medical toxicological and pharmacological input into directing and reporting biochemical toxicology for coroners, supporting the published rationale (Tormey and Moore 2012).

The responses show that there is a clear need for training of coroners to understand the differing roles of medical specialists and the provenance of laboratory scientists. Death certification deserves the same diagnostic standard as antemortem diagnosis.
